# Integrating computational skills in undergraduate Microbiology curricula in developing countries

**DOI:** 10.1093/biomethods/bpad008

**Published:** 2023-06-06

**Authors:** Omolara Adenaike, Olufemi Emmanuel Olabanjo, Ayansewa Adeleke Adedeji

**Affiliations:** Department of Biological Sciences (Microbiology Unit), Oduduwa University, Ipetumodu, Nigeria; Department of Computer Science, Oduduwa University, Ipetumodu, Nigeria; University Library, Oduduwa University, Ipetumodu, Osun State, Nigeria

**Keywords:** bioinformatics, computational skills, curricula, microbiology, R, SPSS, statistical packages, computational biology

## Abstract

The employability of young graduates has gained increasing significance in the labour market of the 21st century. Universities turn out millions of graduates annually, but at the same time, employers highlight their lack of the requisite skills for sustainable employment. We live today in a world of data, and therefore courses that feature numerical and computational tools to gather and analyse data are to be sourced for and integrated into life sciences’ curricula as they provide a number of benefits for both the students and faculty members that are engaged in teaching the courses. The lack of this teaching in undergraduate Microbiology curricula is devastating and leaves a knowledge gap in the graduates that are turned out. This results in an inability of the emerging graduates to compete favourably with their counterparts from other parts of the world. There is a necessity on the part of life science educators to adapt their teaching strategies to best support students’ curricula that prepare them for careers in science. Bioinformatics, Statistics and Programming are key computational skills to embrace by life scientists and the need for training beginning at undergraduate level cannot be overemphasized. This article reviews the need to integrate computational skills in undergraduate Microbiology curricula in developing countries with emphasis on Nigeria.

## Introduction

Microbiologists are currently in an era where biological data are collected at unprecedented volumes using high-throughput technologies. An escalating skill requirement beyond the laboratory and field experiments is to apply computational techniques to find meaningful information from these data [1]. Next-generation sequencing (NGS), which is also named high-throughput sequencing (HTS), provides new ways of detecting microorganisms beyond microbial culture-based methods. The application of NGS in microbiology and medical research is becoming a common practice and has spread rapidly with a profound impact on research, diagnostic and clinical microbiology in recent years, and whole-genome sequencing is becoming the most extensively applied form of NGS. The adoption of NGS for microbiological detection is becoming mainstream, especially for bacteria, viruses, other prokaryotes and fungi [2, 3]. The high-throughput technologies require the development of new methods to manage the data generated by researchers. It is therefore imperative that microbiologists and other life scientists rise on their competence in the management of the data generated from the newly adopted and advancing technologies [4]. There is clearly a gap between the Microbiology curricula of undergraduate courses in our universities today and the requirements for employment in the labour market. Employers are currently looking for and recruiting graduates with skills capable of providing solutions to human problems, reducing human efforts, saving space and time elapsed in the course of providing accurate results to contemporary issues. In Nigeria, in particular, a critical look at the undergraduate Microbiology curricula of most universities suggests an urgent need to reform the curricula and embark upon project designing intervention courses that improve competence [5]. As such, a university graduate with good microbiology results but no additional skills may remain jobless or employed with less pay than a non-graduate with necessary skills to meet the requirements of the recruiting organization. This unfortunate mismatch between qualification and skills possessed vis-a-vis labour market demands affect graduates year after year and this requires urgent attention [6].

In these days of remote working, microbiologists still spend most of the time in the ‘wet lab’, and yet need to provide means to remain relevant and of demand in labour market. Biologists at all levels are well served if they can analyse their data on their own, or at least speak the same technical language as their collaborators who help them with data analysis [7]. The western world and other developed nations have long incorporated biological computing and related tools for analysis, while developing countries are still generally lagging behind. This article provides insight on the need to upgrade undergraduate Microbiology curricula with relevant computer-based skills to enhance employability in the labour market.

## Bioinformatics and computational biology

Bioinformatics can be described as a field of science in which biology, computer science and information technology merge into a single discipline to facilitate biological discoveries. It is the mathematical, statistical and computing methods that aim to solve biological problems using DNA and amino acid sequences and related information [8]. Present-day biological research generates large data, and the collection of the data as well as the interpretation is essential steps in fulfilling the objectives of research. Bioinformatics thereby emerges to achieve the following 3-fold aims: (i) Organization of data in a way to allow access to information and submission of new entries that are produced; (ii) development of tools and resources; and (iii) use of tools to analyse data and interpretation of results in a biological manner useful for research and development [9]. In microbiology, applications of bioinformatics have been used in pathogen identification, detection of virulence factors, resistance analysis and strain typing to mention a few. While it is not necessary for most life science researchers to develop specialist bioinformatic skills (including software development), basic skills in the application of common bioinformatics software and the effective interpretation of results are increasingly required by all life science researchers [10]. There is a need to give attention to build this skill in the career building of today’s life scientists. The learning process should commence with basic courses at undergraduate level, to introduce and instil the necessary skills at an early stage—ultimately, that bioinformatics should be fully integrated into life science degree programmes [11]. Introductory bioinformatics mostly dwells mainly, but extensively, on the different bioinformatics tools, their resources (databases) and methods of applications, such that life scientists not directly involved in a bioinformatics programme can be skilled at basic concepts of bioinformatics tools to avoid misuse and erroneous interpretations of the biological data [12]. Biological databases are libraries of life science information, collected from scientific experiments, published literature, high-throughput experiment technology and computational analyses [13]. Biological databases are generally classified as primary, secondary and composite databases [14]. The purposes of the different types of databases as well as examples of well-known databases, such as GenBank, European Molecular Biology Laboratory, DNA Data Bank of Japan, Protein Data Bank, etc., also form a part of the basic concepts usually studied. Different categories of bioinformatic tools are usually studied in the categories: sequence alignment (homology and similarity tools), protein function analysis, structural analysis as well as sequence analysis [15]. In microbiology and other health-related research, bioinformatics tools and techniques analysing NGS data are increasingly used for the diagnosis and monitoring of infectious diseases [16]. The Basic Local Alignment Search Tool (BLAST), a bioinformatics homology and similarity tool, is an excellent starting point to teach students bioinformatics. The program refers to an algorithm or software used for pairwise sequence alignment. It carries out sequence-similarity searching to find the regions of local similarity to a query sequence. In bioinformatics, the term ‘similarity’ refers to the degree of likeness between two sequences and is expressed as a percentage [17]. Sequence-alignment analysis is one of the most basic and important issues in bioinformatics. Through sequence alignment analysis, the structure and function of biological sequences can further be predicted [18]. Students can be trained to use BLAST to carry out some research activities in microbiology, such as showing that the differences between Gram-positive and Gram-negative bacterial cell envelope structure, which are reflected in the gene content of the genomes of the different bacteria. Also, the determination of bacterial identity can be obtained using bacterial 16S ribosomal RNA sequences, using the computer to access the BLAST site (www.ncbi.nlm.nih.gov/ for Centre for Biotechnology Information website) [19]. Like BLAST, Fast Alignment (FASTA) is usually introduced to students as another fundamental search tool. FASTA also enables the user to rapidly compare a query sequence against large databases and various versions of the program available. BLAST is usually the program of choice due to the relatively greater speed but FASTA has higher sensitivity [20]. For the undergraduate level, studies on biological sequence alignment are usually limited to dual sequence alignment, whereas courses on multiple sequence alignments are taken mostly at postgraduate levels [18]. The Clustal family of algorithms is a group of frequently used tools for multiple sequence alignments in bioinformatics and is therefore not included in the undergraduate curriculum but taught at the higher levels [21].

Computational biology and bioinformatics are often used interchangeably, although these disciplines can be differentiated in various ways despite the overlap. While bioinformatics seeks to develop algorithms, databases, software tools and other computational resources that permit the insightful analysis of biological data, including its acquisition, storage, quantification, annotation, visual exploration and other forms of processing, computational biology seeks to answer specific biological questions using computational strategies. Some computational biology projects develop algorithms and computational tools to analyse biological data for addressing the question of interest, and many computational biology analyses use the tools created by bioinformaticians. The work of many researchers spans both domains [22]. Computational biology, therefore, refers to the use of numerical and computational tools and methods as an alternative or complement to laboratory procedures, in an effort to better answer biological and biomedical questions at a reduced cost. This scientific field heavily overlaps with the field of bioinformatics [23]. The aspects of computational biology that are merged in the introductory bioinformatic courses tend to train students on how to convert a biological question to a computational problem that can be solved using computers. The approaches to solving the computational problems are carried out by writing a solution (algorithm) using a computer program, for example Python. Using this framework, the life science student is able to analyse DNA content, identify protein-binding patterns, compare sequence and discover variation within genomes [24]. Understanding the function of proteins and finding the relationships between the amino acid sequences are important in medicine and several para-medical disciplines. Today, the three-dimensional structures of hundreds of thousands of proteins originating from different organisms are known. These structures are collected in protein databanks. The information provides a better understanding of many biological processes, and is useful in the identification, diagnosis and treatment of different genetic and infectious diseases. Many researchers will arguably need computational biology and bioinformatics to make progress even in the near future [25]. Having contributed to understanding the complexity of diseases, to their mechanisms, systemic behaviours and linkages within an organism as well as epidemiology across populations, these computing skills are now spearheading microbiome research [26]. Therefore, combining substantial knowledge and experience about bioinformatics and biology in a single person would lead to the training of highly skilled and urgently needed scientists [27].

Several literature articles exist today that have demonstrated active learning strategies used successfully to teach bioinformatics and computational biology courses [19, 28, 29]. Cooperative learning of bioinformatics, as proposed by Wright *et al.* [30], involves students working in groups or in pairs. The class exercises are discussed in groups or through think-pair-share, respectively. Kruchten [31] suggested that the bioinformatics module could be included within another course such as General Microbiology or included in the curriculum as a stand-alone-course. According to [24], a feasible teaching method is the approach of interdisciplinary communication and collaboration skills for both life science and computer science students. Mitchell *et al.* [32] reported the adoption of created programme for unifying microbiome analysis applications and associated analysis tutorials to teach and enable students and researchers to effectively analyse real microbiome datasets. Emery and Morgan [33] are of the opinion that the unique method of project-based learning should be explored and encouraged. This is because it promotes active thinking and provides opportunities for interaction and discussion. This method is interesting as it requires initiative and independence from the students. Martins *et al.* [34] reported that the use of tutorial videos as background knowledge before the class practice was beneficial to their students while using bioinformatic tools to identify genes in a genomic sequence and solve genomic-related misconceptions.

A strong bioinformatics development and delivery can be said to be established in Mali through collaborations. Several formal long and short courses on bioinformatics training have been introduced since 2003 through the African Center for Training in Functional Genomics of Insect Vectors of Human Disease, sponsored by WHO as part of the Research and Training in Tropical Diseases initiative. Later on, the West African Center of Excellence for Global Health Bioinformatics Research Training programme was launched in 2017 (Africa). The programme leveraged infrastructure and personnel from: two earlier informatics training programmes, a malaria research project, the University of Sciences, Techniques and Technologies of Bamako, Mali (USTTB) bioinformatics M.Sc. programme and the African Center of Excellence in bioinformatics (ACE) teaching computer laboratories [35]. Jjingo *et al*. [36] reported the pioneering of a 1-year bioinformatics mentorship in Makerere University, Uganda with a robust curriculum splitting into four quarters of 3 months each as shown in Table 1.

**Table 1.** One-year programme competency-based progressive curriculum [36]

Quarter	Course	Major concepts
First quarter	Molecular biology and DNA sequencing (platforms and chemistry) Command line computing and scripting	Central dogma, DNA structure and function, early DNA sequencing methods, HTS methods Linux operating system, Unix file system and navigation, Unix commands and syntax, shell scripting.
Second quarter	Genome assembly and research ethics Genome analysis and genome annotation	De novo and reference-based genome assembly, read quality metrics, genome assembly tools, primary sequence databases, ethics of genomics Workflows for microbial and human genome analysis; read alignment, variant calling and variant annotation
Third quarter	Sequence analysis and phylogenetics Literature review and presentation of scientific writing	Sequence alignment, phylogenetic tools and phylogenetic interpretation Journal clubs; paper reading, paper presentation and paper discussion scientific writing. Manuscript writing; paper arrangement, content and purpose of paper sections, drawing paper figures and graphics
Fourth quarter	Bioinformatics programming and R statistical computing	R studio, R data input/output, R base, management and use of R packages and BioConductor

**Figure 1.**
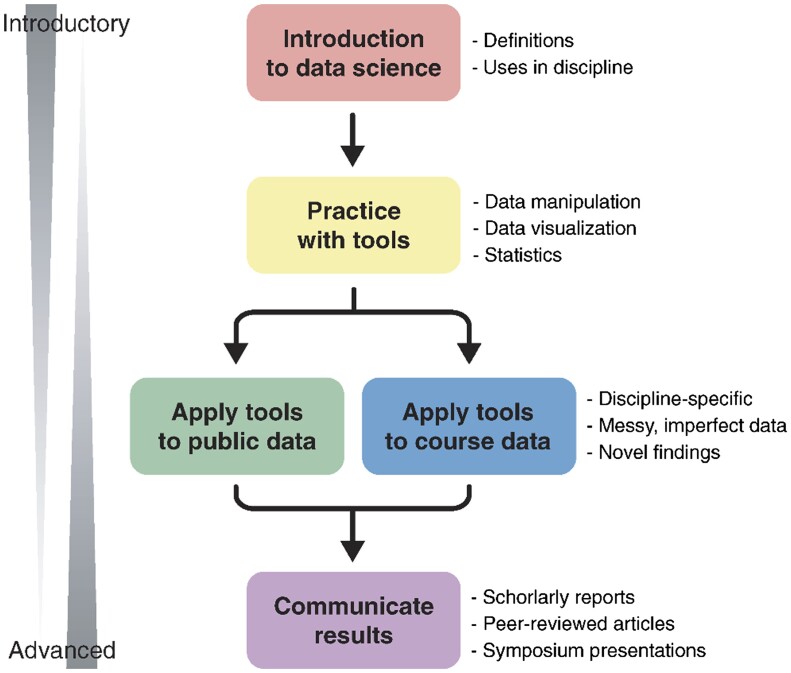
Progressive learning from introductory to advanced data management skill acquisition [39].

It is interesting to note that as a way of advancing the integration of bioinformatics education, several developed countries have introduced it into secondary school education. In the UK, Bain *et al.* [37] reported that for bioinformatics education to keep pace with advances in research, the ‘4273pi Bioinformatics at School Project’ (https://4273pi.org) was designed and delivered curriculum-linked, hands-on bioinformatics workshop for secondary school biology pupils, with an emphasis on equitable access. Over 180 schools were reached in Scotland. The school workshops and teacher training were delivered by a core team of staff and trained volunteers from several universities across the UK. The aim of the project was to provide pupils and teachers with hands-on experience of computing in biology and increase understanding of why it is essential in many areas of life science research. The project was considered to be successful [37]. Educators at California State University, Chico, created a pilot, project-based course in Bioinformatics to address the challenge of insufficient interdisciplinary learning methods for the field. They created a pair of sister courses that introduced biology and computer science students to Bioinformatics separately and in tandem. The two sister courses were ‘Computer Science Bioinformatics’ and ‘Biology Informatics’. Each of the courses was created to teach computer science students the biological approach and to teach biological science students the computational approach to bioinformatics. Students were given assignments and tasks to determine the effectiveness of the curriculum and preparedness for work in the field of Bioinformatics [38]. Dill-McFarland *et al.* [39] reported that, given that data science training is necessary for life science graduates and specialized degree programmes do not attract all students, the University of British Columbia, Canada, proposed progressive learning such that training modules are incorporated into the fabric of undergraduate coursework and students are able to develop confidence and skills in an active learning process that introduces them to the four key aspects of the learning objectives: Introduction; Practice; Application and Communication (Fig. 1).

Integrating bioinformatics education into Microbiology curricula, however, is bound to be challenging in our contemporary educational setting due to the following: first is its cross-disciplinary nature. This means that training and education in bioinformatics would complement knowledge and skills from biology, mathematics, statistics, computer science and information technology. This is a big challenge for trainers. A second reason is the disparate methods, outlooks and cultures of its related disciplines and thirdly, the lack of an integrated training support structure in the form of collaboration for funding and provision of resource persons [40]. This is so because of the need for specific expertise and resources to answer broad research questions with significant scientific impact. Moreso, addressing the challenges associated with the inclusion of multiple disciplines in collaboration will therefore be crucial in life science training programmes [41].

It is noted that setting up and maintaining the appropriate computing infrastructure and developing appropriate learning activities represent major challenges in both adopting and teaching these skills in most universities in Nigeria. Lecturers can be trained or asked to attend short courses or workshops in preparation for this task. Seminars can be arranged to create a diverse community having skills in mathematics, statistics, computer science and biological sciences [42]. These can be adopted as ‘train-the-trainer’ courses, which can go hand-in-hand with providing materials for training at home institutions as well as additional support through e-learning platforms to deal with questions or difficulties when the trained trainers present the course at their home institutions. A group from the same laboratory or institution can be trained in a specific topic or software from time to time [43]. Training for microbiologists can be tailored for relevance and application to topics of interest in the country or region where the training is taking place rather than a broad bioinformatics subject [44].

Worthy of note is the strides of some Nigerian universities in the development of bioinformatics in Nigeria. Covenant University has a bioinformatics research (CUBRe) outfit under the research hub, Covenant University Centre for Research, Innovation and Discovery (CUCRID). CUBRe consists of faculty/graduate staff and students from various science, technology, engineering and STEM disciplines as well as the National Biotechnology Development Agency, Abuja [45]. In 2019, the Centre for Biomedical Science Research of Precious Cornerstone University commenced a 1-month classroom bioinformatics certificate course after an earlier online training module [46]. In 2021, Landmark University, Omu-Aran, Kwara State, hosted a 3-day second Conference of the Nigerian Bioinformatics and Genomics Network (NBGN) [47]. NBGN was inaugurated in June 2019 at the Nigerian Institute of Medical Research, Lagos, Nigeria. This organization aims to advance and sustain the fields of genomics and bioinformatics in Nigeria by serving as a vehicle to foster collaboration, provision of new opportunities for interactions between various interdisciplinary subfields of genomics, computational biology and bioinformatics, as well as providing opportunities for the early career researchers [48]. Other universities can work with NBGN for the training of bioinformatics. The National Open University of Nigeria is noteworthy in offering bioinformatics in undergraduate biological sciences. Several other universities are advancing by offering bioinformatics at the postgraduate level. The introductory aspect should, however, begin at the undergraduate level.

**Figure 2.**
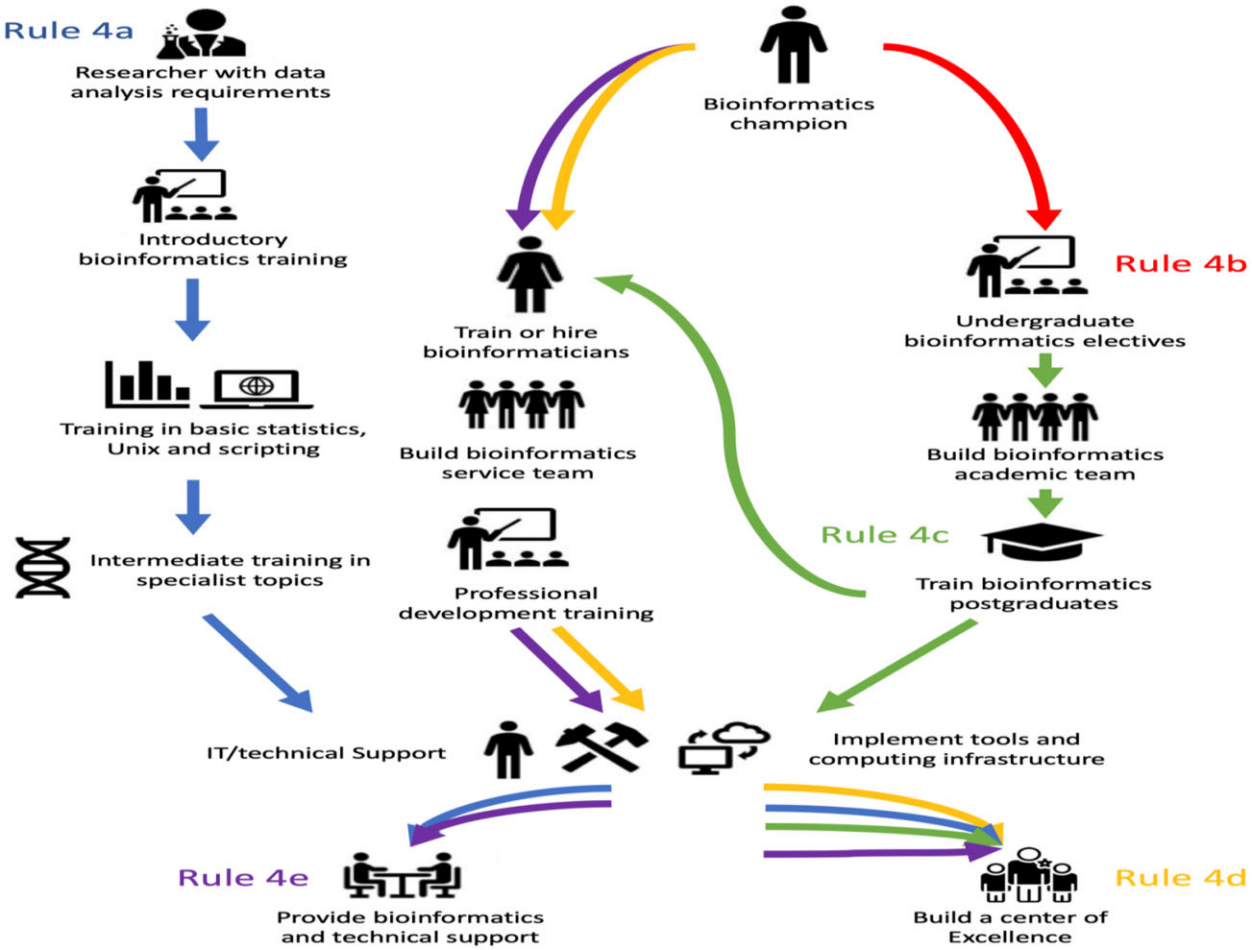
Rule 4 of setting up a bioinformatics capacity at an academic institution [50].

Bioinformatics research efforts in Nigeria have been encouraged over the years with increased support from international scientific organizations for training, research and conference participation. Many Nigerian members of the International Society for Computational Biology have benefitted from such support. Our universities can also utilize these available opportunities [49]. Multi-country organizations such as the H3Africa (Human Heredity and Health in Africa) and H3Africa BioNet (H3ABioNet) consortiums have yielded extensive training and research opportunities within Africa. The H3Africa initiative aims to study genomics and environmental diseases to improve the health of African populations, partnering between the Association of Educational Service Agencies (AESA), the Wellcome Trust, the American Society of Human Genetics (ASHG) and the National Institutes of Health (NIH). The H3Africa Consortium had the effect of diversifying the bioinformatics skills and training in Africa, providing genomics training for over 500 Africans in approximately 5 years. H3ABioNet is a Pan-African bioinformatics network consisting of 32 bioinformatics research groups in 15 African countries and partner institutions in the USA providing bioinformatics training in both introductory bioinformatics topics and specialized topics such as NGS and genome-wide association studies (GWAS) [35]. Aron *et al.* [50], in the paper, ‘Ten simple rules for developing bioinformatics capacity at an academic institution’, proffer detailed explanations on necessary steps. The broadened rule 4 (4a–4e) is graphically presented (Fig. 2). Bioinformatics is recognized as the science of the 21st century and there is therefore a need of time to gain a deeper understanding of the subject, methods and applications for novel discoveries in life sciences that cannot be overemphasized [51].

## Statistical packages (SPSS, R, etc.)

The study and use of statistical sciences in medical and biological sciences have increased rapidly during the last few decades and this aspect is referred to as biostatistics. Therefore, biostatistics is the application of statistics in the field of medicine and biological sciences. It provides powerful tools for creating questions, designing studies, developing measurements and analysing data and has an important place in determining the efficacy and safety of products such as drugs, vaccines, etc. [52]. Therefore, biostatistics has long been a part of the undergraduate microbiology curriculum; however, training in the use of statistical packages as a practical aspect of this course is inadequate in most Nigerian universities. Statistical software (SS) packages are specialized computer programs designed for statistical data analysis. The development of SS in research has transformed the way scientists and researchers conduct their statistical analyses. SS have many useful applications for researchers in the healthcare and biological sciences. Such packages are used to analyse data emanating from a carefully organized scientific process of observation and experimentation. The data analysis can then produce a result that can be interpreted to generate scientific knowledge. The data analysis is often conducted systematically to ensure adequate evaluation of data, and thence describing the data or making statistical inference from the data. The absence of SS or inability to use such packages implies that much scientific data will not transition into knowledge that can guide decision making [53, 54]. A large proportion of students identify statistics courses as the most anxiety-inducing courses in their curriculum [55]. Therefore, teaching statistics in microbiology and other life science programmes should move from the conventional classroom lecture-teaching method to experimental-based teaching, resulting in the acquisition of technical skills and metacognitive analysis which builds up for their personal and professional future [56]. Decades ago, there were not so much data to process, computers available and SS packages for data organization, processing and analysis. Today, the lack of the proper tools available for training is no longer an impediment [57]. SPSS, SAS and R are the most widely used SS packages in biological studies amongst several others [58]. This article will focus on SPSS and R.

### SPSS

SPSS is popularly known as Statistical Package for the Social Sciences or Statistical Product and Service Solutions. SPSS is the widely used SS in social sciences and market analysis. Previously developed by SPSS Inc., it was acquired by IBM in 2009. The software name originally stood for Statistical Package for the Social Sciences, reflecting the original market, although now popular and functional in other fields as well, including biological and health sciences [53, 59]. Several versions of SPSS have been used for analyses in research in microbiology [60–64]. Engaging students in making the important connections between biostatistics and their major disciplines, such as microbiology with SS products like SPSS, can be very helpful. Most students will not pursue additional coursework in biostatistics; as such, the core course provides their only exposure to concepts and methods in biostatistics. Given the brevity of the exposure, we must be realistic about the breadth and depth of the competencies and corresponding curriculum. What level of mastery can reasonably be expected after a one or two semester course(s) in biostatistics? [65]. There is need to intentionally incorporate hands-on training sessions with take-home exercises. This will go a long way in providing a feasible competent mastery level of carefully selected set of statistical procedures as care is taken to limit the list to core areas needed by biological and health sciences. Also, project supervisors should be encouraged to ensure that undergraduate research projects are not statistically deficient. Teaching and training on statistical proficiencies need to be reformulated and one of the most productive steps could be to create courses with an emphasis on interpretation and applications, without reduction in the learning of the theory [66].

### R

R is a statistical and graphical program used frequently in the biological sciences, R is open source, robust and adaptable; it can be used for a range of purposes. R programmers across the world contribute to and iteratively improve R on a daily basis. Thus, it is both broadly used and useful across fields, including biology and industry [67]. R is very flexible and highly customizable, is continuously being developed and the latest version can be downloaded as often as desired. R does require some programming skills, and coding is needed to perform analysis. R SS development environment is designed for data processing, calculation and graphical representation. R therefore offers some advantages including its graphic-visualization capacity and management of large volumes of statistical data. [58]. Many obstacles lie in the way of implementing a curriculum rich in quantitative biology. Teaching R programming to life science students can cause anxiety, especially to new learners and educators alike. Custer *et al.* [68], therefore, developed a self-paced tutorial-module that covers an introduction to basic programming in R and offers students an opportunity to learn the basic skills that often act as a roadblock to learning. The method was effective in changing student attitudes towards learning R as additional time is dedicated to it. Nikitina and Chernukha [69] proposed the possibilities of R programming language in simulating microbiological synthesis processes; they stated that a great number of mathematical models are used to describe kinetics of microbial synthesis, which usually refers to dynamics of substrate consumption, biomass and metabolite production. Several microbiology research examples with R program analysis exist [70–72].

In December 2022, a two-day workshop was conducted for microbiology graduate students at the University of Manitoba to provide an introduction to R programming language functions and to provide some instructions in data visualization and statistical analysis [73]. The benefits of teaching R to undergraduates are numerous. It is recognized as a valuable skillset for undergraduate students, knowing that programming skills can lead to more job opportunities and higher-paying jobs, and are useful across a variety of job categories, microbiology and other life sciences inclusive [67].

R is a ‘new language’ for students, especially for those who never used programming and statistics before, and therefore it should be taught in the introductory phase, simultaneously with basic statistical methods. This helps students make the immediate connection between the statistical methods and the software. If both statistics and R are taught during the first lab section, they become a tool that students are familiar with and will more likely use in the future [74]. Learning R is like learning a new language; it is entering a new world of vocabulary, grammar and syntax, and maybe even a new style of thinking—a lot for students to accommodate. However, R users are fortunate to have an abundance of high-quality published books as learning resources, including over a dozen that are featured on the R Studio website (https://www.rstudio.com/resources/books/), and countless more authored by knowledgeable R contributors [75]. R maintains, and probably will maintain, a niche but dominating presence in the field of bioinformatics, providing ready-made packages and functions [76].

## Programming languages for biological sciences

Computing has revolutionized biological sciences over the past several decades, so much so that virtually all contemporary research in molecular biology, biochemistry and other biosciences, such as microbiology, utilize computer programs [77]. Currently, the most popular use of computer programming languages in biological sciences is in the field of bioinformatics [78]. Bioinformatics does not function in isolation; the effective processing of biological data is accomplished with a variety of programming languages. Many bioinformatics programs or pipelines are coded in languages such as R or rely heavily on Python programming language [79]. It has resulted in an increasing number of universities in the world including bioinformatics, programming or computer science in the curricula of undergraduate microbiology programmes as well as other life sciences, leading to students having understanding of the principal computational concepts underlying the tools they use on a regular basis in their research [80]. This invariably enhances their skills, makes them attractive to employers and invariably provides a good platform for a promising career. Biology-oriented programming classes using Python for students should be given consideration in Nigerian Institutions [81].

Python is a programming language extensively used in bioinformatics and data science, which is particularly suitable for beginners. Python has been described as a suitable method of teaching basic programming language to undergraduate biological students. It is a high-level script programming language, which has been adopted by several universities in the world due to its simple syntax, extensive available documentation and an active developer’s community. Moreover, Python has the Biopython library: a collection of classes, modules and packages with prebuilt functionalities, such as for sequence analysis, genetic of populations, phylogeny, detection of motif regions, biological data visualization, manipulation of protein structures and so on (http://biopython.org). Due to the popularity of Biopython in comparison to other languages, Python has been considered one of the most popular languages for software developing for bioinformatics [82]. Python is an easy-to-learn, versatile and comprehensive tool. It is a good option for everyone—from beginners to experts. Python programming language is used widely in various bioinformatics topics. Python’s simplicity allows bioscientists to concentrate on the problem at hand. They also do not need to spend a lot of time learning the programming language’s syntax or behaviour [83]. Institutions choosing to further the exploration of their students in Python would have a highly-sought-after skill set for a wide variety of data science [84]. Zuvanov [85] described a hands-on and live-coding workshop model for teaching introductory Python programming in Brazil to meet the growing need of acquisition of programming skills for bioscience undergraduate and graduate students alike.

One challenge of teaching these new skills is the effective combination of basic computer-science instruction, statistics and biologically related application. Incorporating this instruction into existing curricula is even more challenging [86]. It can be a process with challenges along the way but often paving a sound path to future success [75].

## Conclusion

In conclusion, this article issues a wakeup call to university curriculum developers and academic planners in developing countries, Nigeria in particular, that the past decades have transformed the world of statistical data analysis, with new methods, new types of data and new computational tools, such that the skills to use these tools must be acquired [87]. Teaching bioinformatics in developing countries like Nigeria can be quite challenging, since it is a multidisciplinary field, where most of the undergraduate and faculty staff have little or no background knowledge or training. However, rendering bioinformatics analysis techniques are the most desirable skills in a variety of employments, scholarship programmes and academic positions [88]. Such skills will improve research in microbiology and increase employability rates and competitiveness in world markets.

The Federal Government of Nigeria’s unveiling of the Core Curriculum Minimum Academic Standards (CCMAS) through the National University Commission (NUC) is quite timely. The guideline provides the Nigerian Universities opportunity to input 30% component of the curriculum with regard to their peculiarities and characteristics [89]. This is a golden opportunity for the institutions to source for courses with contents able to equip their undergraduates as desired with skills that provide competencies in their future career.

Davis *et al.* [90] of the Canadian Society of Microbiologists (CSM) Committee on Microbiology Undergraduate Education proposed the following five examples of curriculum innovation that can be incorporated into microbiology education as follows: (i) data analysis and critical thinking, including ethical discussions at all levels; (ii) a focus on experimental design in the lab; (iii) science communication elements; (iv) a focus on bioinformatics competencies and (v) incorporating equity, diversity and inclusion into the microbiology curriculum.

For a long time, research progress in biological sciences was constricted by the poor yield of data acquisition methods. Today, the great technological progress of the last decades has provided the means for collecting huge data masses with lower cost and effort to analyse and interpret, and Nigeria, as well as other developing countries, must not be left out [91]. Bioinformatics training in low–medium income countries has a lasting positive impact if done well, paying attention to the trio, planning, development and implementation, to tackle the major challenges of shortage of infrastructure, lack of training facilities and poor Internet, lack of local expertise and funding [44].

A conscious joint effort by all stakeholders (i.e. research institutions, governmental entities, teachers, trainers and researchers) is required to deliver and support an action plan that can lead to bioinformatics dissemination in a wider, more structured and cohesive manner in Nigerian Universities and Africa at large [34].
